# 

**Published:** 1905-11-18

**Authors:** 


### NOTICE TO CORRESPONDENTS.

All MSS., Letters, Books for Review, and other matters intended for the Editor should be addressed The Editor, "The Hospital," The Hospital Buildings, 28 & 29 Southampton Street, Strand, W.C.

The Editor cannot undertake to return rejected MS., even when accompanied by stamped directed envelope.

### Notice to Advertisers and Subscribers.

All Advertisements, Orders for Copies of The Hospital, and Business Communications should be addressed to **THE MANAGER (Not to the Editor),** The Hospital, 28 & 29 Southampton Street, Strand, London, W.C.

The Cost of Subscription, post free, is at follows:—Per week, 3½d.; per quarter, 3s. 3d.; per half-year, 6s. 6d.; per annum, 13s. Foreign and Colonial:—Per annum, 19s. 6d., payable, in all cases, in advance.

**NOTICE.—Vol. XXXVII. of "The Hospital" October 1904 to March 1905), in handsome cloth binding, will shortly be on sale at the office of this Journal, price 10s. 6d. Cases for binding the Numbers contained in this Volume 2s. Index to Vol. XXXVII., 3½d., post free.**

**The Monthly Part for November is now ready. Price 1s., by post, 1s. 3d.**

### MEDICAL APPOINTMENTS.

**W. Fraser Annand,** M.D., B.S.Lond., Assistant Resident Medical Officer at Queen Charlotte's Hospital, Marylebone Road, N.W. **Alfred J. Barnes,** L.A.H., M.P.S.I., Examiner in Preliminary Education, Pharmaceutical Society of Ireland. **W. Hirst Bateman,** M.B., Ch.B.Vict., Honorary Medical Officer to the Rochdale Infirmary. **Francis T. Bond,** M.D. Lond., re-appointed Medical Officer of Health to the Thornby District Council, Gloucestershire. **A. Boyle,** M.B.Leeds, Resident Obstetric Officer at the General Infirmary, Leeds. **Harold Fearnley,** M.B.Leeds. Resident Medical Officer at the Leeds Public Dispensary. **A. Gough,** M.B.Leeds, House Physician at the General Infirmary, Leeds. **Alfred Grace** L.S.A.Lond., Deputy Medical Officer for the First District and Workhouse, by the Chipping Sodbury (Gloucestershire) Board of Guardians. **Frank Harvey,** M.R.C.S., L.S.A., Medical Officer to Padstow (Cornwall) Post Office (including St. Merryn and St. Issey), and also Medical Officer to the Padstow and Little Petherick Police. **Harold Leach,** M.R.C.S., L.R.C.P. Lond., House Surgeon at the Hospital for Women and Children, Leeds. **J. H. Legge,** M.B., B.S.Lond., House Surgeon at the General Infirmary, Leeds. **Eva McCall,** M.D.Glasg., Second Assistant Medical Officer at the Hull City Asylum, Willerby. **James Acworth Menzies,** M.D., C.M.Edin., Honorary Ophthalmic Surgeon to the Rochdale Infirmary. **D. Richmond,** M.D., Ch.B.Glasg., Honorary Medical Officer to the Rochdale Infirmary. **W. H. Smailes,** M.B., B.S.Lond., House Surgeon at the General Infirmary, Leeds. **Frederick W. Stewart,** B.A., M.B., R.U.I., Second Assistant Medical Officer to the Kent County Asylum, Barming Heath, near Maidstone. **B. Elinor Walters,** M.B., B.S.Lond., House Surgeon at the York Dispensary. **J. Whalley,** M.B.Leeds, House Physician at the General Infirmary, Leeds.

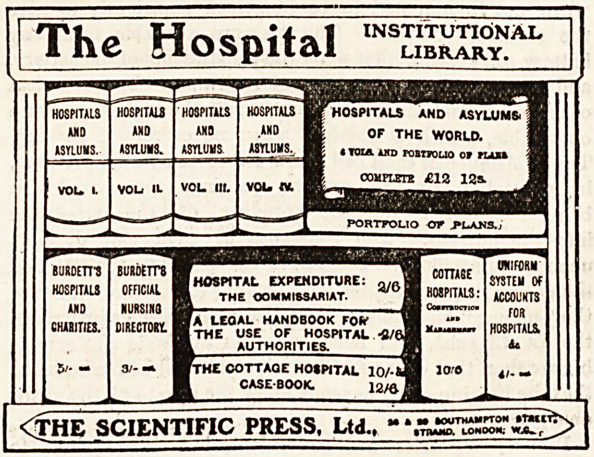

### How to Become a Nurse

Full particulars of Training Schools, Premiums, Salaries, Hours of Duty, and all other details required by anyone desirous of taking up Nursing as a Profession will be found in

**THE NURSING PROFESSION: How and Where to Train.**

By Sir HENRY BURDETT, K.C.B.

Price 2/- net, 2/4 post free.

It is essential to success that anyone taking up Nursing should have some idea of the duties expected of her.

**NURSING HINTS TO PROBATIONERS ON PRACTICAL WORK.**

By ANNESLEY VOYSEY.

Price 2/- net, 2/4 post free.

Contains full particulars of a Nurse's duties and will prove of the greatest assistance to the Probationer.

### A Selection of Books indispensable to the Nurse and Probationer.

Dictionary of Terms used in Medicine, Midwifery and Nursing.

**THE NURSES' PRONOUNCING DICTIONARY.**

By HONNOR MORTEN. New Edition, Revised by MARY I. BURDETT. 2/- post free.

This Dictionary has been used by Nurses for many years, and the fact that it has been recently revised and the phonetic pronunciations added by Miss M. I. Burdett should greatly enhance its value as a work of reference. Its size enables it to be carried comfortably in the apron pocket, which is a strong point in its favour.

How to Bandage.

**PRACTICAL GUIDE TO SURGICAL BANDAGING AND DRESSINGS.**

By WM. JOHNSON SMITH, F.R.C.S. 2/- post free.

A useful guide to Bandaging and Dressing, written especially for Nurses by a surgeon of great experience. The work is up to date and the information thoroughly reliable.

Anatomy and Physiology.

**ELEMENTARY ANATOMY AND SURGERY FOR NURSES.**

By WILLIAM McADAM ECCLES, M.S.Lond., M.B., F.R.C.S., L.R.C.P. 2/6 post free.

**ELEMENTARY PHYSIOLOGY FOR NURSES.**

By C. F. MARSHALL, M.D., B.Sc., F.R.C.S. Crown 8vo, Illustrated, cloth. 2/- post free.

A valuable help to Physiology for Probationers.

Medical and Surgical Nursing.

**A HANDBOOK FOR NURSES.**

By J. K. WATSON, M.D., M.B., C.M. New and Revised Edition. Profusely Illustrated. 5/- net, 5/4 post free.

Medical Electricity.

**MEDICAL ELECTRICITY AND THE LIGHT TREATMENT.**

By KATE NEALE, Sister in Charge Light Department Guy's Hospital. Cloth, profusely Illustrated. 2/6 net, 2/9 post free.

A practical book on a subject hitherto untouched from a nursing standpoint.

A Complete Text-Book of Medical, Surgical, and Monthly Nursing.

**NURSING: ITS THEORY AND PRACTICE.**

By PERCY G. LEWIS, M.D., M.R.C.S., L.S.A. New Edition. Profusely Illustrated. 3/6 post free.

Surgical Work.

**SURGICAL WARD-WORK AND NURSING.**

By ALEXANDER MILES, M.D.Edin., C.M., F.R.C.S.E. Second Edition. With particulars and Illustrations of the Instruments used in various Operations. 3/6 net, 3/10 post free.

**SURGICAL INSTRUMENTS AND APPLIANCES USED IN OPERATIONS.**

A Classified and Illustrated List, with Explanatory Notes. By HAROLD BURROWS, F.R.C.S. Ready Shortly. Limp cloth. 1/6 net, 1/8 post free.

This work will prove of the utmost service as a work of reference to all Nurses, as not only does it contain particulars of instruments but is fully illustrated so that they can be readily recognised.

**THE EXAMINATION OF THE URINE.**

Compiled for the use of Nurses. By J. K. WATSON, M.D., M.B., C.M. 1/- net, 1/1 post free.

**The Scientific Press, Ltd.**

Publishers of Nursing Text-Books, Charts, and Case-Books of all Descriptions.

28 & 29 SOUTHAMPTON STREET, STRAND, LONDON, W.C.

